# Effect of *Porphyromonas gingivalis* lipopolysaccharide administration on non-alcoholic liver disease in Medaka fish

**DOI:** 10.1093/femsmc/xtaf017

**Published:** 2025-11-07

**Authors:** Ayano Ueki, Yukako Ito, Joe Sakamoto, Yasuhiro Kamei, Ayaka Yazawa, Shigeki Kamitani

**Affiliations:** Department of Clinical Nutrition, Graduate School of Comprehensive Rehabilitation, Osaka Prefecture University, Habikino, Osaka 583-8555, Japan; Department of Clinical Nutrition, Graduate School of Comprehensive Rehabilitation, Osaka Prefecture University, Habikino, Osaka 583-8555, Japan; Laboratory for Biothermology, Habikino, Osaka 583-8555, Japan; Laboratory for Biothermology, Habikino, Osaka 583-8555, Japan; Optics and Imaging Facility, National Institute for Basic Biology, Okazaki, Aichi 444-8585, Japan; Department of Basic Biology, School of Life Science, The Graduate University for Advanced Studies (SOKENDAI), Aichi 444-8585, Japan; Department of Clinical Nutrition, Graduate School of Comprehensive Rehabilitation, Osaka Prefecture University, Habikino, Osaka 583-8555, Japan; Department of Nutrition, Graduate School of Human Life and Ecology, Osaka Metropolitan University, Habikino, Osaka 583-8555, Japan; Department of Clinical Nutrition, Graduate School of Comprehensive Rehabilitation, Osaka Prefecture University, Habikino, Osaka 583-8555, Japan; Department of Nutrition, Graduate School of Human Life and Ecology, Osaka Metropolitan University, Habikino, Osaka 583-8555, Japan

**Keywords:** NASH, *Porphyromonas gingivalis*, LPS, medaka

## Abstract

Recently, it has been reported that either infection of rodents with the periodontopathogenic *Porphyromonas gingivalis* (Pg) or administration of its lipopolysaccharide (Pg-LPS) to rodents with non-alcoholic steatohepatitis (NASH) causes progression and exacerbation of the disease. Thus, periodontal disease and NASH are closely related, and further research is required. Medaka (*Oryzias latipes*) has been used as an alternative model for studying human diseases in rodents. In this study, we investigated the association between NASH and Pg-LPS in a NASH model medaka, fed a high-fat diet for 12 weeks, and then injected intraperitoneally with Pg-LPS (low-dose Pg-LPS group: 1.5 mg/kg, high-dose Pg-LPS group: 15 mg/kg) once a week from 5 to 8 weeks. After 12 weeks, the effects of Pg-LPS administration on NASH pathology were evaluated. As a result, liver weight and liver weight/body weight values tended to be higher in the high-dose Pg-LPS group compared to the other groups. HE and Oil Red O staining of the liver showed increased fat accumulation with high-dose Pg-LPS. In addition, Sirius red staining of the liver found fibrosis only in the high-dose Pg-LPS group. These results suggest that Pg-LPS administration may accelerate the progression of the disease in the NASH model medaka.

## Teaser

Pg-LPS administration increased fat accumulation in the liver and changed intestinal microflora in the NAFLD/NASH model of Medaka.

## Introduction

Non-alcoholic fatty liver disease (NAFLD) is a condition that is not caused by other diseases, such as alcohol, viral liver diseases, or drug-induced liver disorders, and is mainly caused by various factors related to metabolic syndrome (Wei et al. 2024). The prevalence of NAFLD is reported to be approximately 30% worldwide, based on surveys conducted between 1990 and 2019 (Younossi et al. 2024). Examining more detailed annual figures, the prevalence rate from 2007 to 2010 and from 2011 to 2015 was approximately 28% in both cases, while the prevalence rate from 2016 to 2019 was 38%, indicating a gradual increase over the years (Younossi et al. 2024). NAFLD can be divided into simple fatty liver, which rarely progresses, and non-alcoholic steatohepatitis (NASH), which causes inflammation and fibrosis of the liver, leading to cirrhosis and liver cancer. The prevalence of NASH in the general population is approximately 5% worldwide, with regional variations reported at 5% in East Asia, 4% in Western Europe, and 5% in North America and Australia (Younossi et al. 2024). In addition, an extensive survey conducted in Japan showed that the prevalence of NASH among subjects was about 3%, and it is estimated that there are more than 2 million NASH patients in Japan(Eguchi et al. 2012). The characteristic pathology of NASH includes fatty degeneration, inflammation, and hepatocellular injury (balloon-like degeneration) (Sheka et al. 2020). Furthermore, hCLS (hepatic crown-like structures) are seen in the progression from NAFLD to NASH (Itoh et al. 2013). hCLS is a histological image of macrophages surrounding, phagocytosing, and processing liver parenchymal cells that have undergone balloon-like degeneration due to excessive fat accumulation. It has been suggested that hCLS may become a biomarker for NASH in the future (Itoh et al. 2013).

The “two-hit theory” (Fernando et al. 2019) has been proposed as a pathogenetic mechanism of NASH/NAFLD. First, fat accumulates in the liver due to metabolic syndrome and insulin resistance, leading to the development of a simple fatty liver (1st hit). Next, oxidative stress and cytokines are added to the liver, which becomes sensitive to external stimuli due to the simple fatty liver, and inflammation and fibrosis are thought to develop (2nd hit). On the other hand, the “multiple parallel hits hypothesis” has been proposed (Tilg et al. 2021), and further investigation of the detailed mechanism is required.

Recently, it has been reported that dysbiosis is closely associated with NASH (Kobayashi et al. 2022). A previous study has shown that bacterial factors are one of the factors involved in the progression of NASH, and LPS, an endotoxin from gram-negative bacteria, has attracted attention (Imajo et al. 2012). Among gram-negative bacteria, LPS from periodontopathogenic bacteria *Porphyromonas gingivalis* (Pg), which is associated with inflammatory diseases caused by bacterial infection, has also been reported to be linked to NASH. It is thought that periodontopathogenic bacteria and their LPS can enter the bloodstream by swallowing oral bacteria or through wounds in the mouth, reaching the whole body and causing inflammation in various organs (Imajo et al. 2014). In addition, the high prevalence of Pg in patients with NASH (Yoneda et al. 2012) and the fact that multiple chronic periodontopathogenic bacteria, including Pg, have been reported to be a risk factor for cirrhosis (Nagao and Tanigawa 2019) and that the progression of liver fibrosis in patients with NAFLD correlates with antibody titers against the type 4 form of the fimA genotype of the Pg lineage gene (Nakahara et al. 2018). In animal studies, a high-fat diet was used to evaluate the effect of Pg on liver fibrosis, and Pg-LPS injected into the gingiva near the first molars of rats fed a high-fat diet caused inflammation of the rats' livers, and the degree of inflammation was more significant than in the group not treated with Pg-LPS (Fujita et al. 2018).

Furthermore, using a Pg-infected mouse model, it was clarified that periodontal pathogens originating from the oral cavity can infect the liver. LPS induces the production of the cell proliferation factor TGF-β1, which causes liver fibrosis (*Nagasaki et al. 2020*). It has also been pointed out that NASH/NAFLD patients have higher blood concentrations of LPS derived from intestinal bacteria than healthy individuals (Harte et al. 2010). Long-term feeding of rats on a high-fat diet has also been shown to increase the inflammatory potential of NASH. It has also been reported that long-term feeding of a high-fat diet to rats causes changes in the intestinal microbiota and increases *Escherichia coli* (*E. coli*)*-derived* LPS and inflammatory cytokines in the serum as the NASH progresses, and that improving the intestinal microbiota can restore intestinal barrier function (Xue et al. 2017) because the hepatic portal vein connects the liver and the intestinal tract. It has also been reported that odontogenic infection with Pg in a high-fat diet (HFD)-induced NASH mouse model resulted in the detection of Pg in the liver and accelerated the progression of neoplastic nodule formation (Sakamoto et al. 2023).

Furthermore, the relationship between the intestinal microbiota and periodontopathogenic bacteria has been investigated in mice fed a high-fat diet, and intravenous injection of a Pg mixture not only progressed NASH but also significantly altered the intestinal microbiota composition. Additionally, it has been reported that administration of Pg-LPS alone does not induce NASH progression in rats; however, when combined with a high-fat diet, it does (*Fujita et al. 2018*). Thus, periodontal disease is closely related to NASH, and further research is required to investigate the relationship between periodontal microbiota and NASH. Additional studies are needed for these findings. Therefore, this study focused on the association between NASH and periodontal disease.

Until now, NASH research has primarily been conducted using rodent models (*Sasaki et al. 2018*). Still, there are issues, such as a lack of models that accurately reflect the clinical condition of patients and the unsuitability for large-scale screening. Additionally, in recent years, from an animal welfare perspective, experiments using mammals in food-related research have become increasingly difficult worldwide (Else 2019). Medaka are as excellent as rodents for drug screening as experimental animals, characterized by high reproductive rates, rapid maturation, and low housing space and daily maintenance costs due to their small size. The medaka genome has been fully sequenced, and techniques for generating transgenic and knockout animals are well-established (Yamauchi et al. 2000, Kasahara et al. 2006). Physiologically, medaka are omnivorous, and their carbohydrate and lipid metabolism patterns resemble those of mammals (Brown and Tappel 1959, Sheridan 1988). However, Matsumoto *et al*. induced NASH in medaka by feeding them a high-fat diet (HFD) for 12 weeks. They confirmed that the progression of liver disease in these fish resembles that of human NASH (Matsumoto et al. 2010). Since NASH is caused solely by excessive lipid intake, the NASH medaka model is considered to be more similar to human patients with NASH. The NASH model medaka fed a high-fat diet showed improvement of the disease when reared in rearing water with diabetes medication (Goto et al. 2019), indicating the usefulness of a simple screening method. However, medaka is small, so dissection and sample collection require skill, and some parameters, such as blood, are challenging to measure. In light of these advantages and disadvantages, if the effects of Pg-LPS can be evaluated using a medaka model of NASH in this study, it will enable large-scale screening at a lower cost and in a smaller space than in mammals and establish a model for the complications of NASH and periodontal disease. In this study, we evaluated the effects of Pg-LPS on the pathogenesis of NASH using a NASH model medaka.

## Materials and methods

### Animals

Production for NAFLD/NASH medaka was performed according to the previous report (*Matsumoto et al. 2010*). Six-month-old Himedaka strain Cab (*Oryzias latipes*) fish, hatched and grown in the laboratory, were used in the experiments. The fish were maintained in a 2 L tank filled with water in an incubator with a 14-hour/10-hour light/dark cycle at a room temperature of 25 ± 1°C, with aeration. The rearing water was changed every two days, and the tanks were cleaned. The fish were divided into three groups (n = 12/group): a PBS group, a low-dose LPS group, and a high-dose LPS group. Each group was fed a standard diet (Hikari Labo M-450, Kyorin, Hyogo, Japan) for one week to acclimate. Then, the fish were fed a high-fat diet (HFD32, CLEA Japan Inc., Tokyo, Japan) at a rate of 20 mg/fish/day for 12 weeks. The PFC ratio and energy per gram (kcal/g) of the diet are shown in Supplemental Table 1. For injection of *Porphyromonas gingivalis* -LPS (Pg-LPS) (InvivoGen, San Diego, CA), fish were anesthetized with fish anesthetic (FA100, Sumitomo Pharma Animal Health Co., Ltd., Osaka, Japan) and received weekly intraperitoneal injections of Pg-LPS from 5 to 8 weeks, starting at week 1 when the high-fat diet was started. The low-dose LPS group received 1.5 mg/kg/week, the high-dose LPS group received 10 µl of Pg-LPS at 15 mg/kg/week, and the PBS group received 10 µl of PBS (-). The dose was based on the previous report (Nakanishi et al. 2019). The fish were weighed under anesthesia at the start of the high-fat diet (week 1) and at the beginning, as well as at 4, 8, and 12 weeks after the diet began. After 4 weeks, two fish in each group were dissected; after 8 and 12 weeks, five fish were dissected. Liver weights were measured, and the liver condition was evaluated by determining the ratio of liver weight to body weight. At the time of dissection, the whole medaka body, the open abdomen, and the liver alone were observed under a stereomicroscope (SZ-40, Olympus, Tokyo, Japan) and photographed using a camera (EOS Kiss X7, Canon, Tokyo, Japan) to evaluate morphological changes. The animal experiment regulations of Osaka Metropolitan University govern the conduct of animal experiments.

### Histology

Livers collected at autopsy were embedded in the Tissue-Tek OCT compound (Sakura Finetek Japan, Tokyo, Japan) and frozen immediately. Frozen tissue was thinly sliced (12 µm) using a rotary microtome (Cryostat HM525NX, PHC Holdings, Tokyo, Japan) and stored at -80°C until staining. Before staining, the tissues were fixed in 4% paraformaldehyde-phosphate buffer (Nacalai Tesque, Kyoto, Japan) for 10 minutes and stained with hematoxylin-eosin (HE) (Muto Pure Chemicals Co. Ltd., Tokyo, Japan), Oil Red O (Muto Pure Chemicals Co. Ltd. Tokyo, Japan), and Sirius Red (Muto Pure Chemicals Co. Ltd. Tokyo, Japan). Oil red O staining was used to evaluate liver fat accumulation, and Sirius red staining was used to assess liver fibrosis (Goto et al. 2019). After staining, the sections were sealed with an oil-based sealant (Histomount, National Diagnostics, Atlanta, GA) for HE and Sirius Red, or a water-soluble sealant (Fluoro-Keeper, Nakalai Tesque, Kyoto, Japan) for Oil Red O. They observed under an optical microscope (CX-23, Olympus, Tokyo, Japan). For all stained tissues, images were photographed using a digital camera (α-7S, Sony, Tokyo, Japan) placed on the microscope at 40x magnification at randomly selected positions.

### Real-time PCR

RNA was extracted from livers (about 30 mg) and immediately frozen after autopsy using an RNA extraction kit (RNeasy Mini Kit; Qiagen, Hilden, Germany). From the extracted total RNA, cDNA was synthesized using a cDNA synthesis kit (Transcriptor First Strand cDNA Synthesis Kit, Roche Diagnostics, Rotkreuz, Switzerland). Synthesized cDNA was mixed with primers and fluorescent dye (MyGo Green Mix Universal ROX, IT-IS Life Science Ltd., Cork, Ireland) for two inflammatory cytokines and nine lipid metabolism-related genes, respectively, and analyzed using a real-time PCR system (qTower³, Analytik Jena, Jena, Germany). According to the manufacturer’s manual, the run protocol was as follows: denaturation was performed at 95°C for 10 seconds, and 40 cycles of 95°C for 10 seconds and 65°C for 25 seconds. The internal standard gene was the 60S ribosomal RNA protein gene (*rpl7*) (Zhang and Hu 2007). The 11 primers were as follows: 1) Tumor Necrosis Factor—alpha (*tnfa*) (Uemura et al. 2015), 2) interleukin 1 beta (*il1b*) (Okamura et al. 2020), 3) Peroxisome proliferator-activated receptor alpha (*ppara*) (Fujisawa et al. 2021), 4) Carnitine palmitoyltransferase 1 (*cpt1*) (Fujisawa et al. 2021), 5) Aconitase 1 (*aco1*) (Oishi et al. 2012), 6) Aconitase 3 (*aco3*) (Oishi et al. 2012), 7) Long-chain acyl-CoA dehydrogenase (*lcad*) (Oishi et al. 2012), 8) Sterol regulatory element binding transcription factor Element Binding Transcription Factor 1 (*srebf1c*) (Fujisawa et al. 2021), 9) Fatty acid synthase (*fasn*) (Fujisawa et al. 2021), 10) Acetyl-CoA carboxylase (*acc1*) (Oishi et al. 2012), 11) apolipoprotein B (*apob*) (Oishi et al. 2012). The primer sequences for each are shown in Supplemental Table 2.

### Evaluation of intestinal bacteria

Genomic DNA was extracted from the intestinal tract, which contained intestinal contents, collected and frozen at autopsy using a DNA extraction kit (DNeasy PowerSoil Pro Kit, Qiagen). The 16S rRNA gene region was amplified by PCR using the extracted DNA as a template. The amplified DNA fragments were purified and sequenced using a next-generation sequencer (Mi-seq, Illumina). Low-quality terminals (quality score < 30) and adapters were removed from the obtained sequence data and denoised using the Deblur plug-in of Qimme2. The database silva-138–99-classifier was used to determine the intestinal bacteria. The alpha diversity index (Shannon index) was calculated, and Bray-Curtis Principal Coordinate Analysis (PCoA) was performed using the Bray-Curtis exponent as a measure of beta diversity.

### Statistical analysis

Body weight, liver weight, body weight-to-liver weight ratio, and real-time PCR were analyzed using one-way ANOVA, and Tukey’s method was employed for multiple comparisons. Prism 9 (Graph Pad) was used for statistical analysis.

## Results

### Apparent Observation

**Figure 1.**
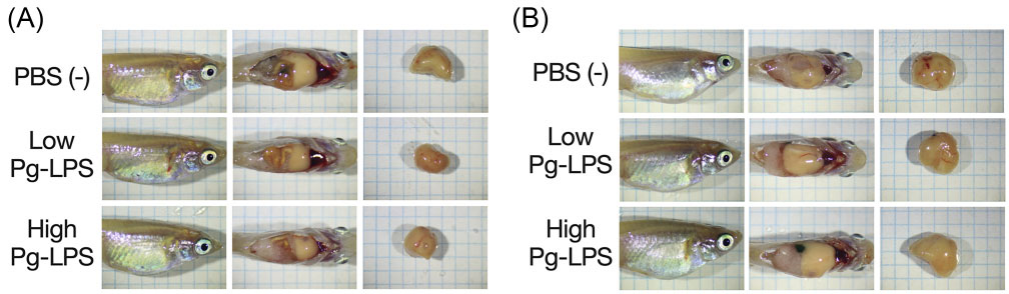
Images of the whole body, open abdomen, and liver of NAFLD/NASH model medaka. Medaka after 8 weeks (A) and 12 weeks (B) of high-fat diet feeding. The length and width of a square on the graph paper are 2 mm.

The whole body, abdominal cavity, and liver of medaka were observed under anesthesia using a stereomicroscope at 4, 8, and 12 weeks after the start of the high-fat diet, and images were captured to evaluate morphological changes. In all groups, the liver showed white discoloration and fat accumulation around the intestines in all weeks (Fig. 1). Compared to 8 weeks after the start of high-fat feeding, the liver was more enlarged at 12 weeks (Fig. 1B). However, no apparent morphological differences were observed between the groups in any of the weeks.

### Body weight, liver weight, and liver weight/body weight

**Figure 2.**
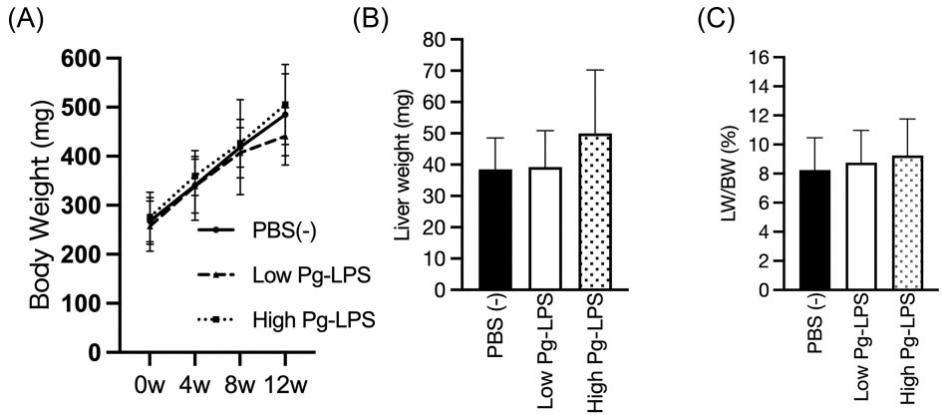
Body weight (BW), Liver weight (LW) and LW/BW. Body weight (BW) (A), Liver weight (B), and LW/BW (C) after 12 weeks of high-fat feeding were shown (n = 5/group). Each data in the graphs represents mean ± SD. One-way ANOVA was used for statistical analysis at *P* < 0.05 significance level, and Tukey’s method was used for multiple comparisons.

Changes in the body weight of medaka during the experiment and liver weight after autopsy were measured. There were no significant differences in body weight (Fig. 2A), liver weight (Fig. 2B), or liver weight/body weight (Fig. 2C) among the groups at any week. However, after 12 weeks, liver weight and liver weight/body weight tended to be higher in the high-dose LPS group than in the other two groups (Fig. 2B, C).

### Pathological changes in the liver of medaka

**Figure 3.**
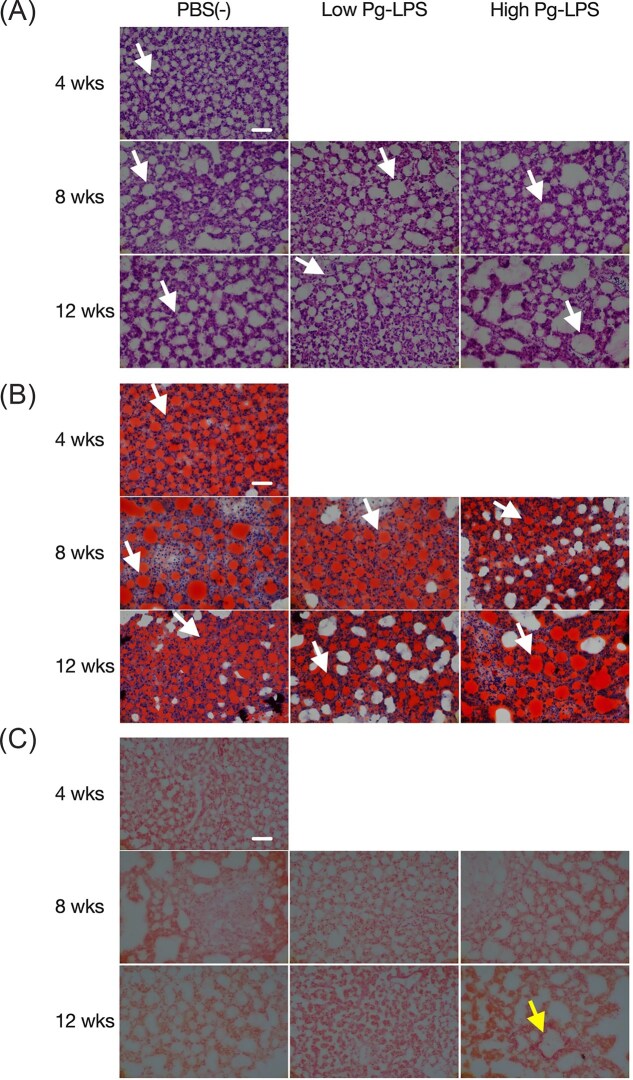
Histopathology of the liver. Pathologically stained images of frozen sections of medaka liver are shown. (A): HE, (B): Oil red O, C: Sirius red. White arrows indicate fat droplets; yellow arrow indicates fibrotic areas. The scale bar indicates 50 μm.

Liver tissue was collected at 4, 8, and 12 weeks after the start of high-fat feeding. Thin tissue sections were prepared and stained with HE for inflammation, Oil Red O for fat accumulation, and Sirius red for fibrosis to evaluate the pathological condition of the liver. Fat droplets were visible in all groups at all weeks (Fig. 3A, B). Compared to 4 weeks, larger fat droplets were observed at 8 weeks and 12 weeks. At 12 weeks, fat droplets in the high-dose LPS group were larger than those in the PBS group (Fig. 3A, B). However, balloon-like degeneration and inflammatory infiltration were not observed in samples collected at 8 and 12 weeks (Fig. 3A). No fibrotic areas were observed in any group at 4 and 8 weeks, or in the PBS group and low-dose LPS group at 12 weeks, but fibrotic areas were observed in the high-dose LPS group at 12 weeks (Fig. 3C).

### Inflammatory Cytokine and Lipid Metabolism-Related Gene Expression

**Figure 4.**
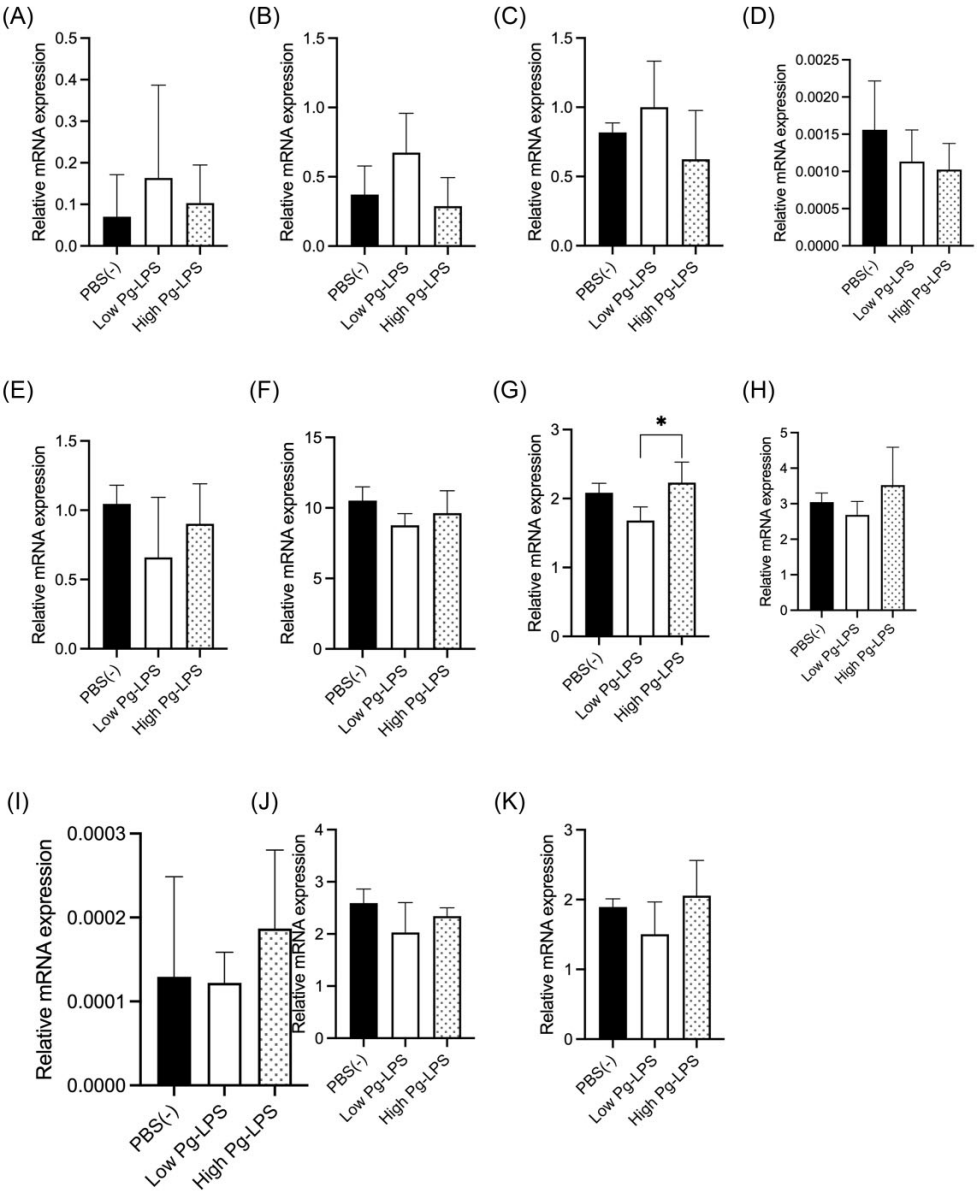
mRNA expression of inflammatory cytokines and lipid metabolism-related genes in the liver 12 1weeks after initiation of high-fat diet feeding. (A): Tumor Necrosis Factor-alpha (*tnfa*), (B): interleukin 1 beta (*il1b*), (C): Peroxisome proliferator-activated receptor-alpha (*ppara*), (D): carnitine palmitoyltransferase 1 (*cpt1*), (E): Aconitase 1 (*ac*o1), (F): Aconitase 3 (*aco3*), (G): Long-chain acyl-CoA dehydrogenase (*lcad*), **(H)**: Sterol Regulatory Element BindingTranscription Factor 1 (*srebf1c*), (I): Fatty acid synthase (*fasn*), (J): Acetyl-CoA carboxylase (*acc1*), (K): apolipoprotein B (*apob*). Each data in the graph represents mean ± SD. Statistical analysis was performed using One-way ANOVA at a significance level of *p* < 0.05 and Tukey for multiple comparisons. *: *P* < 0.05.

Liver samples from medaka, 12 weeks after the initiation of a high-fat diet, were used to evaluate the mRNA expression of inflammatory cytokines and genes related to lipid metabolism (Fig. 4). The genes, *tnfa* and *il1b*, were investigated for inflammatory cytokine production. *srebf1* is the main regulator of fatty acid, *acc1*, and *fasn*. About these gene functions, *ppara* is the regulator of beta-oxidation, and *cpt1, aco3*, and *lcad* are mitochondrial beta-oxidation maker genes, and *aco1* is the peroxisome beta-oxidation maker gene. The gene apo3 codes for the major constituent proteins of lipoproteins, particularly chylomicrons, VLDL, and LDL. Significant differences were observed in the mRNA expression of *lcad* between the low-dose and high-dose LPS groups (Fig. 4G). However, the groups had no significant differences in other genes (Fig. 4).

### Changes in the intestinal microflora of medaka induced by LPS administration

**Figure 5.**
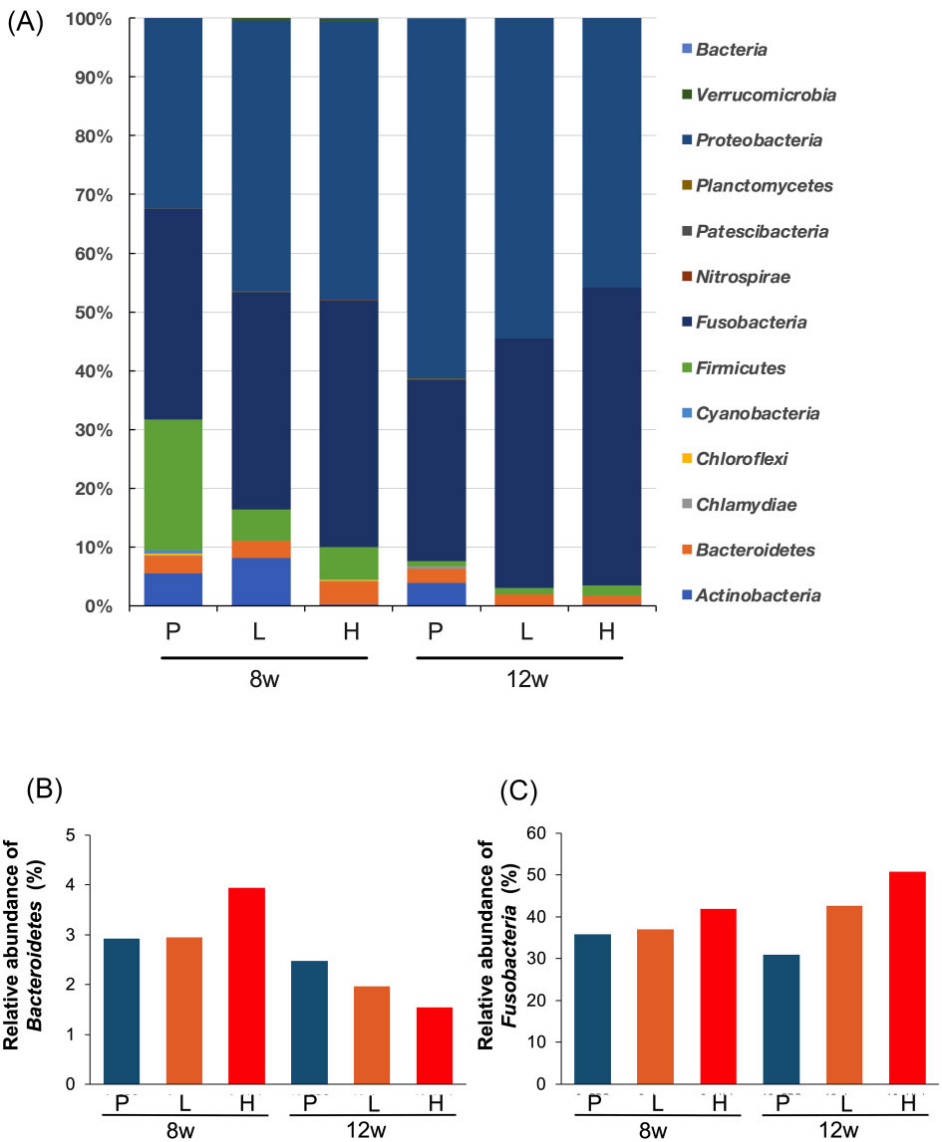
Analysis of the intestinal microflora. (A): Comparison of the composition of the intestinal microflora at the phylum level (showing the percentage of composition when the total of all bacterial species is 100), (B): Relative abundance of the phylum *Bacteroidetes* in the intestinal microflora (%), C: Relative abundance of the phylum *Fusobacteria* in the intestinal bacterial layer (%). P, L, and H indicate the PBS, low-dose LPS, and high-dose LPS groups, respectively.

Intestinal tracts containing intestinal contents collected at autopsy were used to compare the structure of the intestinal microbiota. Occupancy at the phylum level was reduced in all groups at 12 weeks compared to 8 weeks after the start of high-fat diet feeding (Fig. 5A, B). The relative abundance of the *Bacteroidetes* phylum decreased in all groups at 12 weeks compared to 8 weeks (Figs. 5A and B). Comparing the relative abundance of the *Bacteroidetes* phylum in each group after 8 and 12 weeks, the PBS group showed little change. In contrast, the low-dose LPS and high-dose LPS groups exhibited a more pronounced decrease (Figs. 5A, B). The relative abundance of Fusobacteria was higher in the PBS group, followed by the low-dose LPS group and the high-dose LPS group at 8 weeks and 12 weeks (Fig. 5A, C). Comparing the groups at 8 and 12 weeks, the PBS group showed minimal change, whereas the low-dose LPS and high-dose LPS groups exhibited greater increases (Figs. 5A and C). In α-diversity, there was a decrease at 12 weeks compared to 8 weeks (Fig. S1A), and in β-diversity, there was a difference between 8 and 12 weeks (Fig. S1B).

## Discussion

In this study, we investigated the effect of administration of periodontopathogenic bacterium Pg-LPS on the NAFLD/NASH model medaka. When Pg-LPS was administered intraperitoneally to the NAFLD/NASH model medaka, there was no difference in body weight between the groups receiving Pg-LPS. However, there was a tendency for an increase in liver weight and the liver-to-body weight ratio (Fig. 2). Additionally, at 12 weeks, the size of lipid droplets was larger in the high-dose LPS group compared to the PBS group (Figs. 3A, B). This finding suggests that Pg-LPS administration may have enhanced fat accumulation in the liver, leading to an increase in liver weight and the liver-to-body weight ratio.

Among NAFLDs, the simple fatty liver disease rarely progresses beyond fat accumulation in the liver, while NASH shows not only fat accumulation in the liver but also the progression of inflammation and fibrosis of the liver. In this study, fat accumulation in the liver was observed in all groups (Figs. 3A, B). However, fibrosis was only observed in the high-dose LPS group at 12 weeks (Fig. 3C). This result indicates that high-dose LPS administration may be exacerbating the condition. It has already been reported that the intraperitoneal injection of *E. coli*-derived LPS into mice fed a choline-deficient L-amino acid diet causes inflammation, even at low doses, and promotes fat accumulation in the liver, hepatocyte inflammation, and fibrosis (*Nakanishi et al. 2019*). Since a marked increase in TNFα mRNA expression in mouse models fed high-fat diets mixed with LPS from *E. coli* or *Salmonella abortus equi* (Lee et al. 2024), we expected an increase in mRNA expression of proinflammatory cytokines by Pg-LPS administration in the present study. Still, no significant increase was observed (Fig. 4A, B). However, it has been reported that in the zebrafish NAFLD model fed a high-fat diet, TNFα and IL-1β mRNA expression increase compared to a normal diet, even without Pg administration (Zou et al. 2021). Furthermore, TNFα and IL-6 mRNA expression did not increase in high-fat diet-fed mice receiving intravenous Pg (*Sasaki et al. 2018*). Studies using *E. coli*-LPS intraperitoneal injection in high-fat diet mice also show no change in TNFα and IL-6 mRNA expression (*Tanida et al. 2024*). Therefore, the results of this study are likely to be valid. No significant changes were observed between the control and Pg-LPS administration groups in the expression of lipid metabolism-related genes, likely due to the large differences in gene expression levels within each group. (Fig. 4C–K). Regarding the expression of genes involved in lipid metabolism, consistent results have not been obtained even in past papers on medaka NASH models. For example, in the paper by Matsumoto *et al*. (*Matsumoto et al. 2010*), compared to the liver at week 4 of HFD feeding, the genes ACC1, SREBP-1, and FAS, which are related to lipid synthesis, showed increased expression in the liver. Among the genes related to lipid breakdown, CPT1 and PPARα showed decreased expression, while ACO1 showed increased expression. However, in the paper by Torro-Montell *et al*. (Torró-Montell et al. 2019), after 5 days of HFD feeding, FAS and ACC1 showed increased expression, SREBP1 showed no change in expression, and PPFA, ACO1, and CPT1 showed decreased expression. Furthermore, in the paper by Kuwashiro *et al*., after 4 weeks of HFD feeding, FAS showed no change in expression, while ACC1 and PPARγ decreased, ACO1 decreased, and CPT1 increased (Kuwashiro et al. 2011). It is likely that even with the same feeding period, subtle differences in the progression of pathology among individual animals cause such discrepancies in results. These results may be due to differences in food intake resulting from group housing; housing animals individually might yield more accurate data. The reasons for the significant individual differences were (1) the method of rearing and (2) the method of Pg-LPS administration. Regarding the rearing method, it is challenging to feed each medaka an equal amount of food. Since each group of medaka is kept in a single tank, it is challenging to ensure that all medaka consume the same amount of food, which may lead to individual differences in disease status. In addition, it has been recently reported that gender and age are related to the progression of NASH and NAFLD (Yatsuji et al. 2007, Hashimoto and Tokushige 2011) and that they are more common in middle-aged males and older postmenopausal females who become easily obese. Regarding obesity and sex differences, hormonal involvement in the underlying pathophysiology is hypothesized (Lovejoy et al. 2008). In particular, estrogen, one of the female hormones, is known to act centrally to reduce food intake while promoting fat metabolism peripherally(Oosthuyse and Bosch 2011). Consequently, women are more prone to obesity after menopause due to decreased estrogen levels, and it has been noted that fat distribution shifts from subcutaneous to visceral fat (Suzuki and Abdelmalek 2009, Ayonrinde et al. 2011). The present study did not assess the sex of the animals. Regarding the issue of administering Pg-LPS, it is possible that the administration period was too short. In this study, based on a report (Nakanishi et al. 2019) indicating that even low-dose LPS administration significantly affects NASH progression in mice, the dose for the low-dose LPS group was determined, and a dose ten times that amount was administered to the high-dose LPS group. However, although there was a trend toward worsening of NASH after high-dose Pg-LPS administration, no clear difference was observed. It has been reported that treatment of zebrafish larvae with sublethal doses of *E. coli* and *Pseudomonas aeruginosa*-derived LPS, followed by treatment with lethal doses of LPS, did not result in death (Novoa et al. 2009), indicating that fish can become resistant to LPS. Since there are many more bacteria in water than in air, fish may tolerate them to some extent. Therefore, it is considered that administering Pg-LPS for an even longer period may reveal a clear effect on the progression of NASH induced by Pg-LPS administration. Considering the burden on the medaka, a small-volume intraperitoneal administration method was chosen. However, issues remain, such as uncertainty regarding whether the entire dose was administered and the extent to which the intraperitoneally administered Pg-LPS migrated into the tissues. To solve these problems, since dysbiosis of the intestinal microflora and oral microflora has been suggested to be involved in the pathogenesis of NASH (Xue et al. 2017, Sasaki et al. 2018), it is thought that Pg can be administered by feeding vector animals, such as paramecium and artemia that feed on bacteria, and then feeding the vector animals to medaka. Studies have reported the transmission of bacteria infecting zebrafish by feeding them bacteria incorporated into paramecia and artemia (Stones et al. 2017, Chang et al. 2019, Flores et al. 2019). In our laboratory, we have also confirmed via fluorescent imaging of artemia that fluorescently labeled artemia consumes Pg (Sato et al., unpublished data). We are currently investigating a method to feed artemia that consumes Pg to medaka. However, it is not clear how much Pg is preyed on by artemia and how much *E. coli* survives in artemia, although Stones et al. (Stones et al. 2017) reported that *E. coli* was reduced by half in 2–3 hours in a paramecium. Pg-feeding to medaka could reproduce the process by which periodontopathogenic bacteria are introduced into the human body when oral bacteria are swallowed with saliva.

Since it is believed that the progression of NASH is closely linked to the intestinal microbiota, we investigated the effect of Pg-LPS administration on the intestinal microbiota in this study. Comparing the intestinal microbiota composition at the phylum level, we observed a trend toward a decrease in *Bacteroidetes* (Figs. 5A, B) and an increase in *Fusobacteria* (Figs. 5A, C) following the administration of Pg-LPS. It has been reported that NASH patients have significantly decreased *Bacteroidetes* (Mouzaki et al. 2013, Maestri et al. 2022, Xiang et al. 2022) and increased *Fusobacteria* (Rau et al. 2018, Huang et al. 2024), independent of body weight. In the present study, the administration of Pg-LPS may have progressed NAFLD/NASH, resulting in a decrease in *Bacteroidetes* and an increase in *Fusobacteria*. Additionally, a study on the effect of Pg-LPS on dextran sodium sulfate-induced colitis in NASH mice reported a reduction in *Bacteroidetes* (Shen et al. 2021). The *Fusobacteria* phylum is frequently detected in colorectal cancer tissue, and among them, *Fusobacterium varium* has been reported as a possible causative agent of ulcerative colitis (Yukawa et al. 2013, Su et al. 2020). Thus, numerous reports indicate that the *Fusobacteria* phylum is associated with gastrointestinal diseases, primarily in the large intestine. Since the hepatic portal vein connects the liver to the intestinal tract, the pathophysiology of the liver may be significantly influenced by various factors derived from the intestinal tract, including intestinal bacteria, bacterial components, and intestinal bacterial metabolites.

In this study, although no significant differences were observed, administration of Pg-LPS tended to worsen the pathology of the NASH model medaka, and differences were noted in the composition of the gut microbiota. The limitations of this study include the lack of comparison between the data and *E. coli*-LPS administration with Pg-LPS, which causes deterioration of liver pathology. Next, we will investigate this issue to determine which bacteria contribute more to the decline of liver pathology. Now we aim to make the improved NASH model medaka, which is established by Pg feeding to worsen the NAFLD/NASH pathology because patients have simultaneously periodontitis and NASH/NAFLD, which are severe pathologies. Suppose a complication model using medaka can be established. In that case, it will be possible to conduct large-scale screening at lower cost and in less space than with mammals, which is expected to contribute significantly to future research on the functionality of foods.

## Supplementary Material

xtaf017_Supplemental_Files
