# Supplementary material for: Effect of Porphyromonas gingivalis lipopolysaccharide administration on non-alcoholic liver disease in Medaka fish
Source: FEMS Microbes. 2025 Nov 7;6:xtaf017. doi: 10.1093/femsmc/xtaf017 (PMC12641535; doi:10.1093/femsmc/xtaf017)
Supplement: xtaf017_Supplemental_Files [file xtaf017_supplemental_files.zip › FEMSMC-2025-023.R1 one sentence summary.docx]

Pg-LPS administration increased fat accumulation in the liver and changed intestinal microflora in the NAFLD/NASH model of Medaka.
