# Supplementary material for: Effect of Porphyromonas gingivalis lipopolysaccharide administration on non-alcoholic liver disease in Medaka fish
Source: FEMS Microbes. 2025 Nov 7;6:xtaf017. doi: 10.1093/femsmc/xtaf017 (PMC12641535; doi:10.1093/femsmc/xtaf017)
Supplement: xtaf017_Supplemental_Files [file xtaf017_supplemental_files.zip › Figure legends.docx]

**Figure legends**

**Figure 1. Images of the whole body, open abdomen, and liver of NAFLD/NASH model medaka**

Medaka after 8 weeks (A) and 12 weeks (B) of high-fat diet feeding. The length and width of a square on the graph paper are 2 mm.

**Figure 2. Body weight (BW), Liver weight (LW) and LW/BW**

Body weight (BW) (A), Liver weight (B), and LW/BW (C) after 12 weeks of high-fat feeding were shown (n = 5/group). Each data in the graphs represents mean ± SD. One-way ANOVA was used for statistical analysis at p < 0.05 significance level, and Tukey's method was used for multiple comparisons.

**Figure 3. Histopathology of the liver**

Pathologically stained images of frozen sections of medaka liver are shown. A: HE, B: Oil red O, C: Sirius red. White arrows indicate fat droplets; yellow arrows indicate fibrotic areas. The scale bar indicates 50 μm.

**Figure 4. mRNA expression of inflammatory cytokines and lipid metabolism-related genes in the liver 12 1weeks after initiation of high-fat diet feeding**

A: Tumor Necrosis Factor-alpha (*tnfa*), B: interleukin 1 beta (*il1b*), C: Peroxisome proliferator-activated receptor-alpha (*ppara*), D: carnitine palmitoyltransferase 1 (*cpt1*), E: Aconitase 1 (*ac*o1), F: Aconitase 3 (*aco3*), G: Long-chain acyl-CoA dehydrogenase (*lcad*), H: Sterol Regulatory Element BindingTranscription Factor 1 (*srebf1c*), I: Fatty acid synthase (*fasn*), J: Acetyl-CoA carboxylase (*acc1*), K: apolipoprotein B (*apob*). Each data in the graph represents mean ± SD. Statistical analysis was performed using One-way ANOVA at a significance level of p < 0.05 and Tukey for multiple comparisons. *:p < 0.05.

**Figure 5.** **Analysis of the intestinal microflora**

A: Comparison of the composition of the intestinal microflora at the phylum level (showing the percentage of composition when the total of all bacterial species is 100), B: Relative abundance of the phylum *Bacteroidetes* in the intestinal microflora (%), C: Relative abundance of the phylum *Fusobacteria* in the intestinal bacterial layer (%). P, L, and H indicate the PBS, low-dose LPS, and high-dose LPS groups, respectively.
