# Supplementary figures and images for: Effect of Porphyromonas gingivalis lipopolysaccharide administration on non-alcoholic liver disease in Medaka fish
Source: FEMS Microbes. 2025 Nov 7;6:xtaf017. doi: 10.1093/femsmc/xtaf017 (PMC12641535; doi:10.1093/femsmc/xtaf017)

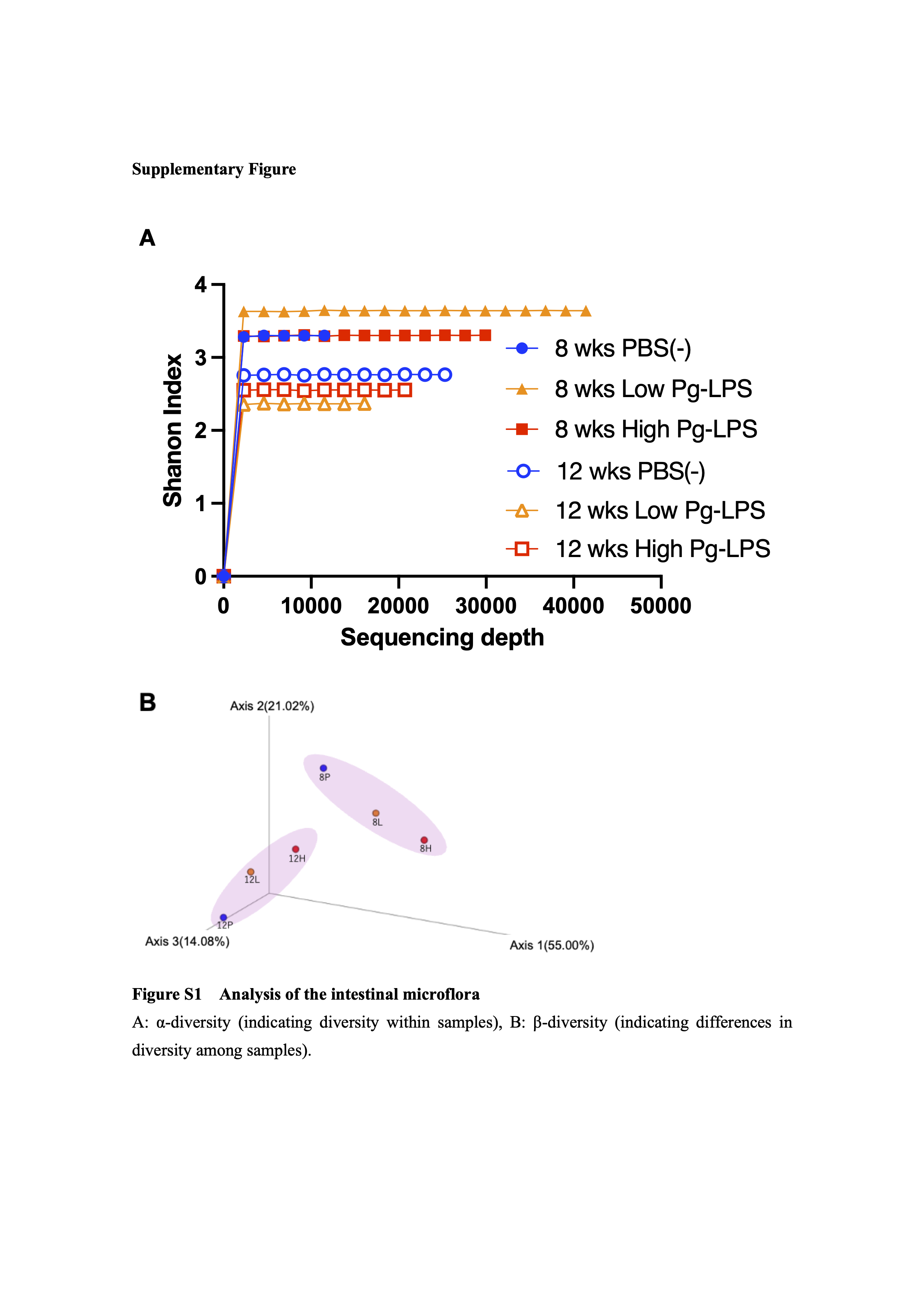

Supplement: xtaf017_Supplemental_Files [file xtaf017_supplemental_files.zip › Supplementary Figure.tiff]

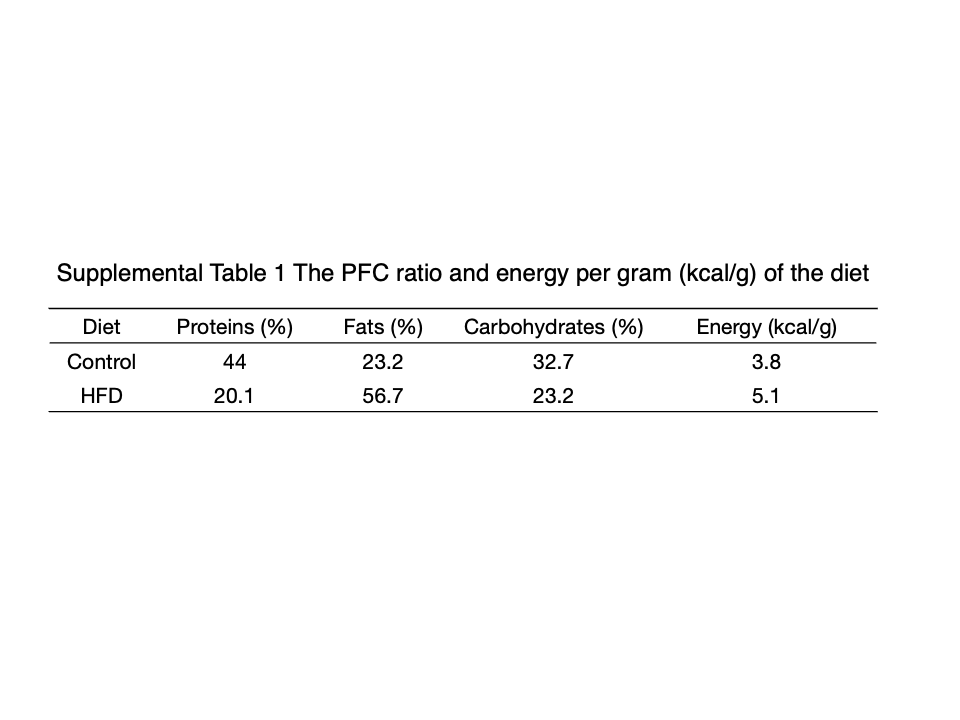

Supplement: xtaf017_Supplemental_Files [file xtaf017_supplemental_files.zip › Table S1.tiff]

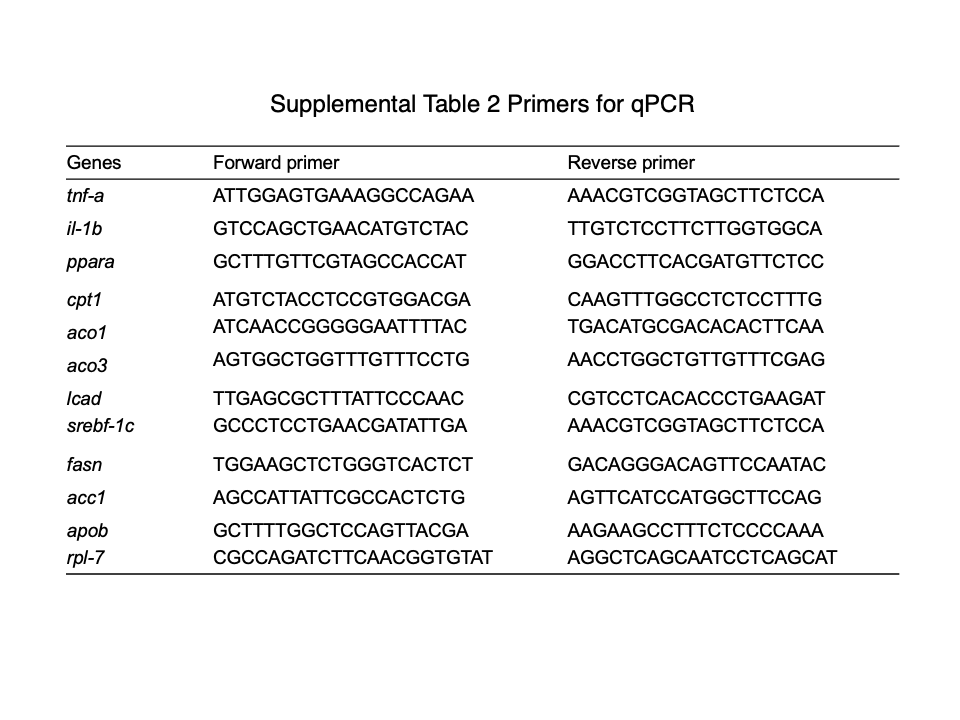

Supplement: xtaf017_Supplemental_Files [file xtaf017_supplemental_files.zip › Table S2.tiff]
