# Supplementary material for: Effect of Porphyromonas gingivalis lipopolysaccharide administration on non-alcoholic liver disease in Medaka fish
Source: FEMS Microbes. 2025 Nov 7;6:xtaf017. doi: 10.1093/femsmc/xtaf017 (PMC12641535; doi:10.1093/femsmc/xtaf017)
Supplement: xtaf017_Supplemental_Files [file xtaf017_supplemental_files.zip › FEMSMC-2025-023.R1 supplementary data - reviews and author response.docx]

**Reviews of manuscript FEMSMC-2025-023**

**Reviewer 1: Noor Salloum, Lawson Health Research Institute, Canadian Centre for Human Microbiome and Probiotics**

**Recommendation: Minor Revision**

The manuscript addresses a timely and relevant topic—the interaction between periodontopathogenic bacteria and NASH pathology—using a medaka model. The study is novel in its use of the medaka fish and the investigation of Pg-LPS effects on hepatic pathology and intestinal microbiota.

However, the manuscript would benefit significantly from improvements in structure, clarity, and the articulation of rationale and results. Below are my comments/recommendations:

Page 4:

Line 12-18: Redundant sentences, combine into 1-2 sentences.

Line 12, at end of ref (2), need a (.)

Line 31 NASH(Itoh… needs a ( )

Line 52: Pg is mentioned first time in the introduction without identification

Line 54: “LPS has also been reported to be associated with NASH” redundant with previous sentence

Line 52, line 55, “LPS.” Should be “LPS”

Page 5:

Line 12: (Nakahara et al. 2018) needs (.)

Line 19: disjointed sentence: LPS induces the production of TGF-β1, a cell growth factor, in NASH/NAFLD patients and causes liver fibrosis (Nagasaki et al. 2020)

Line 48-52: 2 sentences redundant, possibly via paraphrasing

Page 6: The justification for using medaka is present but weakly integrated — it deserves a distinct final paragraph. While the medaka model is presented as advantageous, the rationale for its use over traditional rodent models is weakly explained justifying suitability—especially regarding its physiological relevance to human NASH.

Page 7:

Line 17: including a control group treated with E. coli-derived LPS would strengthen the specificity claim regarding Pg-LPS. At minimum, the lack of this control should be acknowledged as a limitation and discussed (especially since it is mentioned in the discussion that other studies have observed E. coli affect)...

Line 33: discrepancy in the number of dissected fish at different time points needs clarification.

Line 41-42: sentence is incomplete/unclear

Page 8:

Line 21: after (Zhang and Hu 2007), needs a (.)

Line 21: The 11 primers… primers need to be changed into genes/target. Consider adding the function of the target genes for relevancy and explain the relevance of each in the result/discussion section.

Page 9: add statistical analysis for figure 5

Page 10:

Line 10: Four, eight, and 12 weeks, change eight into 8. Consider rephrasing so that the sentence does not start with the word four…

Lines 13-18, consider rephrasing/restructuring the sentence so flows better

Line 28, Figure 2BC, separate B and C with either a (,) or (.), and apply throughout the whole paper… line 36, line 39…

Line 33, clarify that they were liver sections?

Line 33-34, it would be appreciated if the authors reminded what each staining was assessing.

Line 39, separate the sentences for balloon-like degeneration with the sentence related to fibrotic areas, as is, is not too clear.

Page 11:

Line 15-17, sentence structure

Line 17-18, Figure instead of fig for consistency

Page 12:

Line 30-32, discuss and explain why there was the expectation to see and increase, and provide some explanations to why/what is happening so that there was no increase.

most other genes did not show clear trends. Discuss why this would be the only one that shows variability. Discuss potential reasons beyond individual variability, such as sensitivity of qPCR, tissue sampling timing, or low biological effect. What can be done in future studies to resolve issue?

Line 33, individual differences… , what does that refer to.

Line 46, why would middle aged men (in particular)/menopausal women be more prone, vs older men. Would it be feasible to assess this is the lab? Would at the level of the medaka be any observable changes/differences?

Line 47-48, redundant sentence

Page 13:

Line 16-22 redundant sentence, possibly by paraphrasing

Line 24-46 consider splitting into 2 or more sentences…

Line 30, E. coli is not italicised

Line 49 -52 redundant sentences

Figure 5: the trends in Bacteroidetes and Fusobacteria are mentioned, no statistical significance is shown. discuss and clarify whether these changes are statistically supported.

Page 14:

Line 9: Please clarify what constitutes a “complication-like model” of NASH and periodontal disease. What specific adjustments (e.g., route of administration, duration, dosing) are needed to achieve this? How can this model suggested be accomplished. what needs to be done, and differently so that it gains the complication title?

Line 12-13: Suppose we can establish a complication model using medaka., sentence unclear

Page 20: consider adding a visual on the figure itself for size of each square

Page 22, figure 3: legend mentions yellow and white arrows for fibrotic and fat droplets, respectively, but not seen on figure. Please update the figure or revise the legend.

Page 23: figure 4: list the name of the gene on the axis or on too of each gene/target for ease

Page 24: figure 5. B, C name the bacteria on the axis or on top for ease.

statistical analysis?

Figures 4 and 5 would be more informative if gene and bacteria names, respectively, were added to the axes or a title on top, for easier interpretation.

It might be worth using a dot chart, where the individual data points are shown (to help understand the variation in sample values) in addition to the bars representing the mean.

**Reviewer 2**

**Recommendation: Major Revision**

The manuscript describes a potential alternate to a rat model in assessing liver disease, which appears to be a sound approach. Unfortunately for the project, there were no significant findings. I believe that the trends that are seen in the data are overstated throughout the paper and should be addressed to moderate the language used. The discussion goes into a multitude of potential reasons for the neutral results, which should allow for a much more robust follow up study. In the mean time, the current manuscript is still viable if the results are discussed and interpreted with the view of it being a model in development that needs refinement to tease out the small trends seen here.

In general, the manuscript would also benefit from proofreading, as there are many sentences that could be worded better. Some are pointed out in the attached detailed list.

Attached document:

pg4, line 12 - is the (2) supposed to be a reference? If not, it needs to be clearer where the numbers come from (Younossi 2024?)

pg4, line51-52 - probably should redefine LPS and Pg separately from having done it in the abstract.

pg4, line 52 - what is the disease? Pg is the infectious organism, which causes inflammation. This passage needs to be clairified, and the extraneous periods removed after 'LPS' on line 52 and 55.

Pg5, line 6-12 – that is a long sentence that doesn’t go anywhere after the reference.

Pg5, lines 49-52 – sentence is repeated

Pg7, line 21 – PFC should be defined

Pg7, line 41 – the last sentence is incomplete – is there an ethics number, or some other description/clarification that should be there?

Pg7, line 46 – what compound?

Pg7, line 58 – “After staining,[?…..?]sealed with an oil-based sealant…”

Pg10, line 48 – how significant is the result if it non-significantly falls either side of the control?

Pg12, line 12 – I’m not particularly comfortable with the indication that weights changed with diet. Especially the LW/BW measure, which is such a tiny change relative to the error bars.

Pg12, line 16 – how do you account for the apparently large fat drops in the Control at 8 weeks (fig 3B)?

Pg12, line 17 – I don’t know that the results in figs 2 and 3 support this statement

Pg13, line 30 – italicize *E. coli*

Pg13, line 40 – do you have a theory behind the high *Bacteroidetes* abundance in the high dose group at 8 weeks before the mentioned decrease? (higher than the control -> lower than the control)

Figure 3 – I’m not seeing any annotations. This is quite important given how similar the panels look with regard to cell morphology etc

Fig 4 – Please fix the legend to remove the annotations from SILVA so that only the Phylum names are there. If possible, also make the differentiation of the groups clearer – there are only 6 or 7 phyla that are abundant enough to see a colour anyway.

Fig S1a – Two groups are labelled 12wk low

Table S1 – I assume carbonate should be carbohydrate?

**Associate Editor Recommendation: Minor Revision**

While one reviewer has recommended a significant revision, the requested changes from both reviewers primarily focus on presentation, clarity, and moderation of interpretation, rather than on generating new data or altering the core methodology. The scientific approach is sound, and the manuscript remains viable in its current form, provided adjustments are made to the conclusions, the rationale and methods are clarified, and the figure/table presentations are improved. These revisions can be addressed without the need for additional experiments, and the core findings and conclusions remain intact. Accordingly, the decision is minor revision, with the expectation that the authors address the detailed points raised by both reviewers.

**EIC Decision: Minor Revision**

**Author's Response to Decision Letter for (FEMSMC-2025-023)**

Dear Editor,

We thank you for the opportunity to revise the paper for FEMS microbe. We are submitting herewith the revised manuscript entitled "Effect of Porphyromonas gingivalis lipopolysaccharide (Pg-LPS) Administration on NAFLD/NASH Medaka." We have carefully read all the comments from the reviewers and the Editor's decision. We have included a point-to-point Reply to the reviewers' comments. Changes in the revised manuscript were indicated by yellow highlighting in the revised version of the text.

Additionally, our campus relocated to a new location in Osaka City this summer. Thus, my corresponding address was changed.

We hope the revised manuscript is now acceptable for publication in Pathogens as a research article.

Yours sincerely,

Shigeki Kamitani, Ph.D.

Professor

Department of Nutrition, Graduate School of Human Life and Ecology, Osaka Metropolitan University

Zip code: 536-8525, 2-1-132 Morinomiya, Joto-ku, Osaka, Japan

Tel:06-6167-1310 (+81-6-6167-1310)

E-mail: skami@omu.ac.jp

Reply to the reviewers' comments.

Response to Reviewer 1

Thank you for your comments and suggestions. The following are point-to-point responses. Changes in the text are highlighted in yellow.

Reviewer: 1

Comments to the Author

The manuscript addresses a timely and relevant topic—the interaction between periodontopathogenic bacteria and NASH pathology—using a medaka model. The study is novel in its use of the medaka fish and the investigation of Pg-LPS effects on hepatic pathology and intestinal microbiota. However, the manuscript would benefit significantly from improvements in structure, clarity, and the articulation of rationale and results. Below are my comments/recommendations:

1. Page 4: Line 12-18: Redundant sentences, combine into 1-2 sentences.

Authors' responses

We organized and reconstructed the sentences in this portion.

2. Page 4: Line 12, at end of ref (2), need a (.)

Authors' responses

We apologize for the mistyping. We added the period.

3. Page 4: Line 31 NASH (Itoh… needs a ( )

Authors' responses

We apologize for the mistyping. We added the space.

4. Page 4: Pg is mentioned for the first time in the introduction without identification

Authors' responses

We add the full name in the first-time description at the introduction (Page 3).

5. Page 4: Line 54: "LPS has also been reported to be associated with NASH" redundant with previous sentence.

Authors' responses

We omitted the sentence.

6. Page 4: Line 52, line 55, "LPS." Should be "LPS"

Authors' responses

We apologize for the mistyping. We omitted the period.

7. Page 5: Line 12: (Nakahara et al. 2018) needs (.)

Authors' responses

We apologize for the mistyping. We added the period.

8. Page 5: Line 19: disjointed sentence: LPS induces the production of TGF-β1, a cell growth factor, in NASH/NAFLD patients and causes liver fibrosis (Nagasaki et al. 2020)

Authors' responses

We organized and reconstructed the sentences in this portion.

9. Page 5: Line 48-52: 2 sentences redundant, possibly via paraphrasing

Authors' responses

We organized and reconstructed the sentences in this portion.

10. Page 6: The justification for using medaka is present but weakly integrated — it deserves a distinct final paragraph. While the medaka model is presented as advantageous, the rationale for its use over traditional rodent models is weakly explained justifying suitability—especially regarding its physiological relevance to human NASH.

Authors' responses

We added the sentences and references that explain the physiological relevance of medaka to human NASH.

11. Page 7: Line 17: including a control group treated with E. coli-derived LPS would strengthen the specificity claim regarding Pg-LPS. At minimum, the lack of this control should be acknowledged as a limitation and discussed (especially since it is mentioned in the discussion that other studies have observed E. coli affect)...

Authors' responses

We agreed with the reviewer's comment. However, this is the first attempt to investigate the effect of Pg-LPS on the NAFLD/NASH medaka model, building on previous studies using rodents. We will attempt a comparison of Pg-LPS and E. coli-LPS in the future. Thus, we add the sentences that mention the limitations of this study.

12. Page 7: Line 33: discrepancy in the number of dissected fish at different time points needs clarification.

Authors' responses

The medaka at week 4 after HFD feeding were dissected immediately before LPS administration, representing their pre-treatment state. Since this study compared weeks 8 and 12 post-LPS administration, only two individuals were dissected and analyzed at week 4.

13. Page 7: Line 41-42: sentence is incomplete/unclear

Authors' responses

We apologize for the mistyping. The sentence is completed.

14. Page 8: after (Zhang and Hu 2007), needs a (.)

Authors' responses

We apologize for the mistyping. We added the period.

15. Page 8: Line 21: The 11 primers… primers need to be changed into genes/target. Consider adding the function of the target genes for relevancy and explain the relevance of each in the result/discussion section.

Authors' responses

We have added a brief explanation of each gene to the results, as per the reviewer's suggestion.

16. Page 9: add statistical analysis for figure 5

Authors' responses

Unfortunately, since the amount of intestinal contents was considered insufficiently small, the intestinal contents from each medaka group were combined into a single sample for analysis; therefore, no statistical analysis was performed.

17. Page 10: Line 10: Four, eight, and 12 weeks, change eight into 8. Consider rephrasing so that the sentence does not start with the word four…

Authors' responses

We rewrote the sentences according to the reviewer's suggestion.

18. Page 10: Lines 13-18, consider rephrasing/restructuring the sentence so flows better

Authors' responses

We rewrote the sentences according to the reviewer's suggestion.

19. Page 10: Line 28, Figure 2BC, separate B and C with either a (,) or (.), and apply throughout the whole paper…

Authors' responses

We rewrote the sentences according to the reviewer's suggestion.

20. Page 10: line 36, line 39…

Authors' responses

We rewrote the sentences according to the reviewer's suggestion.

21. Page 10: Line 33, clarify that they were liver sections?

Authors' responses

We rewrote the sentences according to the reviewer's suggestion.

22. Page 10: Line 33-34, it would be appreciated if the authors reminded what each staining was assessing.

Authors' responses

We rewrote the sentences according to the reviewer's suggestion.

23. Page 10: Line 39, separate the sentences for balloon-like degeneration with the sentence related to fibrotic areas, as is, is not too clear.

Authors' responses

We rewrote the sentences according to the reviewer's suggestion.

24. Page 11: Line 15-17, sentence structure

Authors' responses

We rewrote the sentences according to the reviewer's suggestion.

25. Page 11: Line 17-18, Figure instead of fig for consistency

Authors' responses

We apologize for the mistyping. We collected the word.

26. Page 12: Line 30-32, discuss and explain why there was the expectation to see and increase, and provide some explanations to why/what is happening so that there was no increase.

most other genes did not show clear trends. Discuss why this would be the only one that shows variability. Discuss potential reasons beyond individual variability, such as sensitivity of qPCR, tissue sampling timing, or low biological effect. What can be done in future studies to resolve issue?

Authors' responses

The expectation that Pg administration would increase inflammatory cytokine gene expression stemmed from reports, such as one showing a marked increase in TNFα mRNA expression in mouse models fed high-fat diets mixed with LPS from E. coli or Salmonella abortus equi (Lee et al., 2024). However, it has been reported that in the zebrafish NAFLD model fed a high-fat diet, TNFα and IL-1β mRNA expression increase compared to a normal diet, even without Pg administration (Zou et al. 2021). Furthermore, TNFα and IL-6 mRNA expression did not increase in high-fat diet-fed mice receiving intravenous Pg (Sasaki et al., 2018). Studies using E. coli-LPS intraperitoneal injection in high-fat diet mice also show no change in TNFα and IL-6 mRNA expression (Tanida et al., 2024). Therefore, the results of this study are likely to be valid. No significant changes were observed between the control and Pg-LPS administration groups in the expression of lipid metabolism-related genes, likely due to the large differences in gene expression levels within each group. (Figure 4C-4K). Regarding the expression of genes involved in lipid metabolism, consistent results have not been obtained even in past papers on medaka NASH models. For example, in the paper by Matsumoto et al. (Mat,sumoto et al., 2010), compared to the liver at week 4 of HFD feeding, the genes ACC1, SREBP-1, and FAS, which are related to lipid synthesis, showed increased liver expression. Among the genes related to lipid breakdown, CPT1 and PPARα showed decreased expression, while ACO1 showed increased expression. However, in the paper by Torro-Montell et al. (Torró-Montell et al, 2019, after 5 days of HFD feeding, FAS and ACC1 showed increased expression, SREBP1 showed no change in expression, and PPFA, ACO1, and CPT1 showed decreased expression. Furthermore, in the paper by Kuwashiro et al., after 4 weeks of HFD feeding, FAS showed no change in expression, while ACC1 and PPARγ decreased, ACO1 decreased, and CPT1 increased (Kuwashiro et al., 2011). It is likely that even with the same feeding period, subtle differences in the progression of pathology among individual animals cause such discrepancies in results. These results may be due to differences in food intake resulting from group housing; housing animals individually might yield more accurate data. We organized and reconstructed the sentences, including those in the above reports, in this portion.

27. Page 12: Line 33, individual differences… , what does that refer to.

Authors' responses

We organized and reconstructed the sentences in this portion.

28. Page 12: Line 46, why would middle aged men (in particular)/menopausal women be more prone, vs older men. Would it be feasible to assess this is the lab? Would at the level of the medaka be any observable changes/differences?

Authors' responses

They are more common in middle-aged males and older postmenopausal females who become easily obese. Regarding obesity and sex differences, hormonal involvement in the underlying pathophysiology is hypothesized. In particular, estrogen, one of the female hormones, is known to act centrally to reduce food intake while promoting fat metabolism peripherally. Consequently, women are more prone to obesity after menopause due to decreased estrogen levels, and it has been noted that fat distribution shifts from subcutaneous to visceral fat. Some previous studies investigated the physical situation of female medaka with OVX operation, like menopausal women. We added the sentences to explain above.

29. Page 12: Line 47-48, redundant sentence

Authors' responses

We organized and reconstructed the sentences in this portion

30. Page 13: Line 16-22 redundant sentence, possibly by paraphrasing

Authors' responses

We organized and reconstructed the sentences in this portion.

31. Page 13: Line 24-46 consider splitting into 2 or more sentences…

Authors' responses

We organized and reconstructed the sentences in this portion.

32. Page 13: Line 30, E. coli is not italicized

Authors' responses

We apologize for the mistyping.

33. Page 13: Line 49 -52 redundant sentences

Authors' responses

We organized and reconstructed the sentences in this portion.

34. Page 13: Figure 5: the trends in Bacteroidetes and Fusobacteria are mentioned, no statistical significance is shown. discuss and clarify whether these changes are statistically supported.

9

Authors' responses

Unfortunately, since the amount of intestinal contents was considered insufficiently small, the intestinal contents from each medaka group were combined into a single sample for analysis; therefore, no statistical analysis was performed. However, since these results represent the average values for each group, they are considered to minimize differences between individuals, allowing us to discern a consistent trend regarding the relative abundance of gut microbiota.

35. Page 14: Line 9: Please clarify what constitutes a "complication-like model" of NASH and periodontal disease. What specific adjustments (e.g., route of administration, duration, dosing) are needed to achieve this? How can this model suggested be accomplished. what needs to be done, and differently so that it gains the complication title?

Authors' responses

We apologize for confusing the reviewer due to our poor description. Now we aim to make the improved NASH model medaka, which is established by Pg feeding to worsen the NAFLD/NASH pathology because patients have simultaneously periodontitis and NASH/NAFLD, which are severe pathologies. Thus, we organized and reconstructed the sentences in this portion.

36. Page 14: Lines 12-13: Suppose we can establish a complication model using medaka. The sentence is unclear.

Authors' responses

We organized and reconstructed the sentences in this portion.

37. Page 20: consider adding a visual on the figure itself for size of each square.

Authors' responses

We show a scale bar inside the images at each 4-week data point. In the Figure legend of Figure 3, the scale bar indicates 50 μm.

38. Page 22, figure 3: legend mentions yellow and white arrows for fibrotic and fat droplets, respectively, but not seen on figure. Please update the figure or revise the legend.

Authors' responses

We apologize for the misdrawing. The arrows disappeared during conversion to TIFF images. We replaced the images with the correct ones.

39. Page 23: figure 4: list the name of the gene on the axis or on too of each gene/target for ease.

Authors' responses

We added the gene names to the axis.

40. Page 24: figure 5. B, C name the bacteria on the axis or on top for ease.

statistical analysis?

Authors' responses

We added the bacteria's names to the axis. Unfortunately, since the amount of intestinal contents was considered insufficiently small, the intestinal contents from each medaka group were combined into a single sample for analysis; therefore, no statistical analysis was performed.

41. Figures 4 and 5 would be more informative if the gene and bacteria names, respectively, were added to the axes or a title above, for easier interpretation.

It might be worth using a dot chart, where the individual data points are shown (to help understand the variation in sample values) in addition to the bars representing the mean.

Authors' responses

We added the gene and bacterial names to the axis and changed the graph style according to the reviewer's suggestion.

Reply to the reviewers' comments.

Response to Reviewer 2

Thank you for your comments and suggestions. The following are point-to-point responses. Changes in the text are highlighted in yellow.

Reviewer: 2

Comments to the Author

The manuscript describes a potential alternate to a rat model in assessing liver disease, which appears to be a sound approach. Unfortunately for the project, there were no significant findings. I believe that the trends that are seen in the data are overstated throughout the paper and should be addressed to moderate the language used. The discussion goes into a multitude of potential reasons for the neutral results, which should allow for a much more robust follow up study. In the mean time, the current manuscript is still viable if the results are discussed and interpreted with the view of it being a model in development that needs refinement to tease out the small trends seen here.

In general, the manuscript would also benefit from proofreading, as there are many sentences that could be worded better. Some are pointed out in the attached detailed list.

Authors' responses

According to the reviewer's suggestion, we have thoroughly restructured the main text and performed a detailed proofreading. Furthermore, we have carefully considered the results, noting that no significant worsening was observed following the intraperitoneal injection of Pg-LPS, and have accordingly modified the wording of the conclusions.

**Reviews of manuscript FEMSMC-2025-023.R1**

**Reviewer 1: Noor Salloum, Lawson Health Research Institute, Canadian Centre for Human Microbiome and Probiotics**

**Recommendation: Accept**

Thank you for addressing the comments and for the substantial improvements made to the manuscript. The revised version is much clearer and more comprehensible. I have no further comments at this stage.

**Reviewer 2**

**Recommendation: Accept**

The revisions made to the manuscript in response to the comments have been a nice improvement. I had provided a separate document with a list of additional comments (on top of those responded to above). Many were addressed in response to comments from the other reviewer, but the following should still be addressed. Since they are very minor, further review after these changes is not needed.

Table S1 – I assume carbonate should be carbohydrate?

Fig S1a – Two groups are labelled 12wk low

Fig 4 – Please fix the legend to remove the annotations from SILVA so that only the Phylum names are there. If possible, also make the differentiation of the groups clearer – there are only 6 or 7 phyla that are abundant enough to see a colour anyway.

**Associate Editor Recommendation: Accept**

**EIC Decision: Accept**
